# Comparative Analysis of Pretreatment Methods for High-Content Red Kidney Bean Bread: Effects on Processing Characteristics and Bread Quality

**DOI:** 10.3390/foods15173051

**Published:** 2026-08-28

**Authors:** Jiajia Zhao, Xiangting Hou, Tingting Li, Waleed Al-Ansi, Mingcong Fan, Yan Li, Haifeng Qian, Li Wang

**Affiliations:** 1 Culinary Science and Technology Department, Jiangsu College of Tourism, 88 Yuxiu Road, Yangzhou 225000, China; zhaojj@jstc.edu.cn; 2 State Key Laboratory of Food Science and Resources, School of Food Science and Technology, National Engineering Research Center for Functional Food, Jiangnan University, 1800 Lihu Avenue, Wuxi 214122, China; ig_houxiangting@cofco.com (X.H.); fanmc@jiangnan.edu.cn (M.F.); liyan0520@jiangnan.edu.cn (Y.L.); qianhaifeng@jiangnan.edu.cn (H.Q.); 3 Department of Food Science and Engineering, College of Light Industry and Food Engineering, Nanjing Forestry University, Nanjing 210037, China; litingting@njfu.edu.cn; 4 Faculty of Agricultural, Environmental and Food Sciences, Free University of Bozen-Bolzano, 39012 Bolzano, Italy; walansi@unibz.it

**Keywords:** red kidney beans, legume-enriched bread, hydrothermal treatment, dough structure, bread quality

## Abstract

Food processing can modify legume structure and improve its applicability in protein-fortified foods. This study investigated the effects of six pretreatments, including peeling, roasting, atmospheric steaming, high-pressure steaming, sprouting, and microwaving, on the processing characteristics and quality attributes of wheat bread containing 50% red kidney bean flour (RKBF). Results showed that thermal pretreatments reduced the gelatinization enthalpy (ΔH) of composite flours by 4.6–13.2% and increased dough water absorption rate by 5.7–9.7% compared with untreated RKBF. Hydrothermal treatments significantly improved dough rheology and structural integrity by increasing glutenin macropolymer (GMP) content, reducing free sulfhydryl levels, altering protein secondary structure, and optimizing starch and gluten distribution. Atmospheric steaming increased the bread specific volume from 3.37 to 3.53 cm^3^/g and reduced hardness by 16%. Incorporation of RKBF also reduced rapidly digestible starch from 64.91% in wheat bread to 48.99–56.16% and increased resistant starch from 11.76% to 21.27–25.55%. Moreover, volatile analysis identified 42 compounds, with thermal pretreatments effectively reducing beany flavor compounds. Peeling alleviated dark coloration but further reduced bread volume, whereas sprouting-induced protein degradation adversely affected dough formation and gluten structure. Overall, atmospheric steaming exhibited the best balance between processing performance and nutritional quality, showing a significant improvement in the bread quality containing 50% RKBF.

## 1. Introduction

Bread is one of the world’s most widely consumed staple foods, with global per capita bread consumption ranging from 59 to 70 kg per year [1]. However, refined wheat flour, the primary component of bread, is low in protein, fiber, minerals, and bioactive compounds. It is also deficient in the essential amino acid lysine, which limits protein digestion and absorption [2]. It has been reported that more than one-third of the global population is still suffering from malnutrition [3] and nearly one billion people are chronically protein deficient [4]. As a result, it is critical to select an appropriate ingredient for the nutritional fortification of wheat flour.

Incorporating natural, high-protein plant-based ingredients into wheat flour represents an excellent strategy for enriching its nutritional profile. Legumes, due to their nutritional benefits (high in protein, fiber, minerals, and phytochemical content), as well as their affordability and accessibility, are desirable ingredients for food producers. Red kidney bean (*Phaseolus vulgaris* L.) is an underappreciated legume with potential for nutritional fortification in wheat-based products. It is high in protein (22.06–32.63 g/100 g wet basis), starch (25.33–49.7 g/100 g dry basis), and dietary fiber (16.21–24.50 g/100 g dry basis) [5]. It also possesses an essential amino acid (EAA) pattern that is compatible with the World Health Organization’s guidelines [6], making it an excellent nutritional supplement to grain proteins [7]. Furthermore, red kidney beans provide multiple health benefits, including anti-obesity, anti-aging, and anti-diabetic activities [8,9,10]. Therefore, incorporating red kidney beans into wheat-based products could be a beneficial approach to enhance their nutritional and functional attributes.

Currently, high-content legume flour substitution is only employed in a few bakery products, such as some pasta and cookie snacks [11], which have no strict gluten-related requirements. However, for bread production, only small amounts (typically less than 10%) of legume flour can be added to wheat bread recipes without impairing their technical and sensory qualities, which are essential factors in customer acceptability [12,13]. Since legume proteins are deficient in gluten-forming gliadin and glutenin, incorporating large amounts of legume flour into wheat flour significantly degrades the dough’s structure and rheological properties, thus impacting the overall quality of the final bread [14]. In addition to the technical drawbacks, the undesirable beany flavor associated with legume flour also considerably influences consumer acceptance [15,16].

Prior to milling, various procedures such as soaking, peeling, roasting, cooking, steaming, sprouting, and fermentation have been proposed to modify the functional properties of legume flour. These pretreatments also influence the nutrient profile and quality attributes of bread [17,18]. Peeling treatment improves the digestibility and palatability of legume flour [19]. The changes in chemical composition and biological activity of some enzymes during germination can improve the technical and functional characteristics of seeds [20]. Heat treatment is also considered an effective pretreatment method as it induces protein denaturation [21,22], which reduces the solubility and functional properties of the proteins, thereby reducing interference with gluten development [23]. In addition, these treatments can further improve the nutritional value and sensory attributes of legume bread by eliminating anti-nutritional factors and removing the beany flavor [24,25].

Different types of legume flour have been incorporated into wheat bread at varying substitution levels. For example, pretreated green pea flour has been used to replace wheat flour at levels ranging from 10% to 50%. Its incorporation can increase the protein and dietary fiber contents of bread, although higher substitution levels may significantly affect dough rheological properties and bread quality [26]. Similarly, germinated Bambara groundnut flour has been used as a partial substitute for wheat flour, increasing the contents of dietary fiber, minerals, resistant starch, slowly digestible starch, and phenolic compounds, while also affecting dough water absorption and mixing properties. A substitution level of 20% was reported to provide the best overall bread quality and sensory acceptability [27]. In addition, non-germinated and germinated alfalfa seed flours have been incorporated at substitution levels of 5% and 10%, demonstrating potential for improving the nutritional composition and antioxidant properties of wheat bread [28]. Overall, the incorporation levels of legume flours reported in previous studies range from approximately 5% to 50%. However, increasing the substitution level may adversely affect gluten network development, dough rheological behavior, and texture. Therefore, appropriate pretreatment strategies to improve the technological suitability of legume flours at relatively high substitution levels warrant further investigation. In this paper, to further enhance the nutritional quality of legume flour-fortified bread, the addition of bean flour was increased to 50%, and the effects of six different pretreatments (peeling, roasting, atmospheric steaming, high-pressure steaming, sprouting, and microwaving) on the processing (gelatinization and mixing properties), structural (dynamic rheology, protein secondary structure, glutenin macropolymer, free sulfhydryl groups, and microstructure), sensory (volume, texture, pore structure, color, and volatile components), and nutritional (amino acid composition and in vitro starch digestion) properties were investigated. These results help to understand how pretreatment can optimize the characteristics of red kidney bean dough, making it more suitable for use in the production of baked goods such as bread. For industrial purposes, selecting an optimal pretreatment method may enhance product quality and market competitiveness.

## 2. Materials and Methods

### 2.1. Raw Materials

Red kidney beans (moisture 7.86%, crude protein 27.90%, starch 28.06%, crude fat 1.30%) were purchased from Xinzhou, Shanxi; gluten flour (moisture 9.21, protein 83.40%, starch 6.69%, crude fat 0.7%), was purchased from Shandong Qufeng Food Technology Co., Ltd. (Weifang, China); high-gluten wheat flour (moisture 11.08%, protein 15.32%, starch 72.0%, crude fat 1.6%), was purchased from Dongguan Yihai Kerry Grain and Oil Food Industry Co., Ltd. (Dongguan, China).

### 2.2. Pretreatments of Red Kidney Beans

Peeling: Red kidney beans were soaked in water overnight at 4 °C, then peeled.

Roasting: Beans were baked at 160 °C in an oven (WTNS36, United Wistron Machinery Co., Ltd., Wuxi, China) for 20 min.

Atmospheric steaming: Beans were steamed in a Midea steamer (MZ-SYH28-2A, Midea Electric Co., Ltd., Foshan, China) for 30 min.

High-pressure steaming: Beans were steamed at 121 °C for 15 min in an autoclave (GI-54DWS, Hede Technology Co., Ltd., Beijing, China).

Sprouting: Beans were spread on plastic trays and incubated at 37 °C with 80% humidity for three days for germination using a constant-temperature incubator (BMJ-250C, Boxun Medical Biological Instrument Co., Ltd., Shanghai, China).

Microwaving: A 125 g portion of beans (for one batch of bread production) was microwaved (M1-L236A, Midea Electric Co., Ltd., Foshan, China) in a glass dish at 800 W for 2 min.

Untreated and pretreated red kidney beans were air-dried at room temperature to about 8% moisture, then powdered using a flour mill (HAINA, Wuhan, China) at 1600 W for 20 s, three times, and sieved through an 80-mesh sieve (180 μm), with the above ones repeating the powdering process, and sealed for storage in a drying apparatus containing anhydrous copper sulfate until needed.

### 2.3. Starch Gelatinization

The gelatinization properties of the sample were assessed using a DSC 3 calorimeter (Mettler-Toledo GmbH, Zurich, Switzerland). A sample mass ranging from 2.0 mg to 5.0 mg was utilized, with a sample-to-distilled water ratio of 1:3. Prior to measurement, the sample crucible was equilibrated at 4 °C for 24 h. A blank crucible served as a control, and the heating program involved increasing the temperature from 20 °C to 120 °C at a rate of 10 °C/min.

### 2.4. Mixing Properties

The mixing properties of red kidney bean dough (RKBD) were assessed using a Mixolab 2 (Chopin Technologies Pty Ltd., Paris, France) [29]. The appropriate sample amount was calculated based on the flour’s moisture content to ensure that the dough reached a weight of 75 g and achieved a torque of 1.1 ± 0.05 Nm. The test was conducted according to the Chopin+ protocol in the Mixolab software (version 4.00), which involves running the test at 30 °C for 8 min, then heating up to 90 °C at a rate of 4 °C per min, maintaining 90 °C for 7 min, then cooling down to 50 °C at a rate of 4 °C per min, and running for an additional 5 min.

### 2.5. Dough Preparation and Structural Properties Test

#### 2.5.1. Dough Preparation

The RKBD was prepared according to the previous study [30] and consisted of 250 g of mixed grain flour based on red kidney bean flour (RKBF) (50% RKBF, 37.5% high-gluten flour, and 12.5% gluten flour), 2.0% dry yeast, 8.0% white sugar, 15.0% butter, and an optimal amount of water (about 80%). The WB consisted of 250 g high-gluten flour, 2.0% dry yeast, 8.0% white sugar, 15.0% butter, and an optimal amount of water (about 65%). The flour mix and dry ingredients were poured into the mixer (WTN-25 from Joint Wistron, WuXi, China), and the dough was obtained by slowly adding water at a low speed for 3 min at 20 °C, then switching to high speed for 2 min until the dough was well formed. Subsequently, butter was incorporated and kneaded at low speed until optimal gluten development was achieved. Additionally, a fresh RKBD without dry yeast was prepared and divided into two parts: one for dynamic rheological testing, and the other for freeze-drying and pulverization (80 mesh) for other analytical experiments.

#### 2.5.2. Dynamic Rheological Properties

A DHR-3 rheometer (Waters Co., Milford, MA, USA) was used to evaluate RKBD’s dynamic rheology. The samples’ linear viscoelastic range was measured using strain sweep testing (0.01–20%) at a frequency of 1 Hz with parallel plates 40 mm in diameter and separated by a 1 mm gap. Subsequently, a frequency sweep test was performed at 25 °C using a strain of 0.02%, and the frequency sweep ranged from 0.1 to 100 rad/s. The measurements included energy storage modulus (G′), loss modulus (G″), and loss tangent (tan δ).

#### 2.5.3. Fourier Transform Infrared Spectroscopy (FT IR)

A Nicolet IS10 FT-IR spectrometer (Thermo Nicolet Corporation, Madison, WI, USA) was used to determine the protein secondary structure of RKBD. Each sample underwent 64 scans within the spectral range of 400 to 4000 cm^−1^, utilizing a resolution of 4 cm^−1^ for each scan. The spectra between 1600 and 1700 cm^−1^ were fitted with Fourier deconvolution and second-order derivatives using PeakFit 4.12 software. The subpeaks from Fourier deconvolution and second-order derivatives usually numbered 7, and we estimated the percentage distribution of secondary structures by calculating the area of subpeaks distributed in different wavelength bands.

#### 2.5.4. Glutenin Macropolymer (GMP) and Free Sulfhydryl Groups (SHf) Content

The GMP content was determined using a modified version of a previously reported method [30]. Specifically, 0.3 g of freeze-dried dough powder was uniformly suspended in 3 mL of 1.5% (*w*/*v*) sodium dodecyl sulfate (SDS) solution and agitated at room temperature for 12 h. The suspension was then centrifuged at 10,000× *g* for 20 min, after which the supernatant was discarded. The protein content in the precipitate was quantified using the Kjeldahl method, and the GMP content was subsequently calculated using the following formula.

(1) $$GMP\;content\left(\%\right)=\frac{GMP\;content}{Crude\;protein\;content\;of\;blend}\times 100$$

The procedure for quantifying free sulfhydryl content was adapted from a previously reported method with some modifications [31]. A 50 mg dough sample was accurately weighed and dissolved in 5 mL of Tris-glycine buffer (containing 10.4 g/L Tris, 6.9 g/L glycine, 1.2 g/L EDTA, 0.5% SDS, 8 M urea, with the pH adjusted to 8.0). The mixture was then subjected to shaking at 25 °C and 180 rpm for 30 min. Afterward, the mixture was centrifuged at 8000 rpm for 5 min. One milliliter of the supernatant was taken, and 0.05 mL of Ellman’s reagent (4 mg/mL of 5,5′-dithiobis-(2-nitrobenzoic acid) dissolved in buffer) was added. The mixture was immediately vortexed and allowed to react in the dark for 30 min, and then the absorbance was measured at 412 nm.

(2) $$SH(\mu mol/g)=\frac{\left(73.53\times A_{412}\times D\right)}{C}$$

#### 2.5.5. Confocal Laser Scanning Microscopy (CLSM) Analysis

Fresh dough samples were cut into 20 μm-thick sections using a cryosectioner (CM1850 UV, Leica, Wetzlar, Germany). The sections were then stained for 30 s with a solution containing 0.025% (*w*/*w*) rhodopsin B (dissolved in distilled water) and 0.25% (*w*/*w*) fluorescein 5-isothiocyanate (dissolved in acetone). After shielding the slices from light and rinsing off any excess dye with deionized water, images were captured at a resolution of 1024 × 1024 pixels using CLSM (LSM880, Carl Zeiss AG, Oberkochen, Germany), and ZEN 2012 software (version 8.1, Carl Zeiss Microscopy GmbH, Oberkochen, Germany) was utilized for processing.

### 2.6. Bread Preparation and Physicochemical Properties Test

#### 2.6.1. Preparation of Red Kidney Bean Bread (RKBB)

The dough was rested at room temperature for 20 min, split into sections of 120 g, shaped and placed in molds (L × W × H: 85 × 85 × 75 mm), and left to rise for 60 min at 37 ± 2 °C and 80 ± 5% rh (WTN-L18SP, Union Weichuang Machinery Co., Ltd., Wuxi, China). To prepare the RKBB, the dough was placed in the oven (WTN-536, Union Weichuang Machinery Co., Ltd., Wuxi, China) and baked at 190 °C on the top and 180 °C on the bottom for 20 min.

#### 2.6.2. Baking Characteristics

The specific volume was determined by the volume-to-mass ratio measured using the millet substitution method. The L*, a*, and b* values of both the bread crust and crumb were investigated utilizing a colorimeter (TES-135A, Tes Electrical Electronic Corp., Taibei, China) [32]. After cooling for 2 h, the bread was cut into uniformly thin slices (15 mm), with six slices taken for TPA testing using a texture analyzer (TA.XTC-18, Baosheng Industrial Development Co., Shanghai, China) equipped with a 20 kg load cell. The measured TPA parameters included hardness, springiness, cohesiveness, chewiness, and resilience. A P36R probe was used with the following parameters: downward compression rate of 2 mm/s, measurement rate of 1.5 mm/s, strain of 5 g, deformation of 50%, and compression interval of 4 s. The bread sections were scanned with a scanner (IR-ADV 4225, Cano Scan LiDE110, Tokyo, Japan), and pore properties were analyzed using ImageJ software (version 1.54h, NIH, Bethesda, USA).

#### 2.6.3. Amino Acid Composition

The total amino acid (TAA) content in the freeze-dried RKBB was quantified by HPLC (Agilent 1260, Agilent Technologies, Palo Alto, CA, USA) with both acidic and alkaline hydrolysis. For acid hydrolysis, the RKBB was treated with 6 mol/L HCl at 110 °C for 22–24 h, then neutralized using 10 mol/L NaOH and diluted to 25 mL with distilled water. For alkaline hydrolysis, RKBB underwent treatment with 10 mol/L NaOH followed by 6 mol/L HCl. The resulting hydrolysates were filtered and centrifuged at 4 °C and 10,000× *g* for 10 min to obtain the supernatants. Chromatographic conditions included a Hypersil ODS C18 column (4.6 mm × 150 mm, 5 μm), operated at a temperature of 40 °C with detection wavelengths of 338 nm and 262 nm. The mobile phases were 20 mmol/L sodium acetate (A) and 20 mmol/L sodium acetate: methanol: acetonitrile = 1:2:2 (*v*/*v*) (B), flowing at a rate of 1.0 mL/min.

The essential amino acid index (EAAI) was estimated as follows:

(3) $$EAAI=\sqrt[{n}]{\prod_{i=1}^{n}\left(\frac{A_{i}}{B_{i}}\times 100\right)}$$

where A_i_ represents the concentration of EAA in the sample (mg/g) and B_i_ represents the concentration of EAA in the reference egg protein (mg/g) [32].

#### 2.6.4. In Vitro Starch Digestion

The starch digestibility was assessed using the previously outlined methods [33]. Bread samples were sieved through an 80-mesh sieve after freeze-drying at −15 °C for 48 h. Samples (0.6 g) together with 6 glass beads were placed into 10 mL of pepsin solution and shaken in a water bath (180 rpm, 37 °C) for 1 h. Then, 10 mL of sodium acetate buffer (pH = 5.2, 0.2 M) and 5 mL of enzyme mixture (trypsin + amyloglucosidase) were added to each tube. After 0, 5, 10, 20, 30, 45, 60, 90, 120, 150, and 180 min of digestion, a volume of the digested solution (0.1 mL) was transferred to a pre-filled centrifuge tube containing anhydrous ethanol (0.9 mL) to stop the digestion process. The mixture was then immediately centrifuged at 8000× *g* for 5 min. The freeze-dried bread samples were analyzed for total starch (TS) content using a colorimetric method (Total Starch Assay Kit, Megazyme, Bray, Ireland), and the glucose content of the supernatant was determined by the glucose oxidase method. The hydrolysis rate (%) and the levels of rapidly digestible starch (RDS), slowly digestible starch (SDS), and resistant starch (RS) were determined using the following equations:

(4) $$Hydrolysis\;rate\left(\%\right)=\frac{G_{t}\times 0.9}{TS}\times 100\%$$

(5) $$RDS\left(\%\right)=\frac{(G_{20}-G_{0})\times 0.9}{TS}\times 100\%$$

(6) $$SDS\left(\%\right)=\frac{(G_{120}-G_{20})\times 0.9}{TS}\times 100\%$$

(7) $$RS\left(\%\right)=\frac{(TS-RDS-SDS)\times 0.9}{TS}\times 100\%$$

where G_t_ represents the amount of glucose released into the system during a specific period.

The starch hydrolysis curve was described using a first-order kinetic equation [34]:

(8) $$C_{t}=C_{\infty }(1-e^{-kt})$$

where C_t_ (%) represents the digestibility of starch at time t (min), C∞(%) denotes the final digestibility of starch, and k (min^−1^) estimates the rate constant for starch digestibility.

The estimated glycemic index (eGI) value was calculated using the following formula:

(9) eGI=0.862×HI+8.198

#### 2.6.5. Volatile Compounds

GC-MS (Agilent 8890-5977B, Agilent Technologies, USA) with an SH-WAX capillary column (30 m × 0.25 mm × 0.25 μm, Shimadzu, Kyoto, Japen) was used to investigate the volatile components in RKBB [35]. RKBB (3 g) was added to a 20 mL headspace vial, and adsorption was conducted at 60 °C for 30 min using an aged extraction head. The detector and injection port temperatures were set to 250 °C, and the adsorbed extractor was then desorbed at 250 °C for 3 min in the gas chromatographic column inlet. The column temperature was initially set at 40 °C for 3 min, then increased linearly from 40 to 90 °C at a rate of 5 °C/min, and finally to 250 °C at a rate of 10 °C/min for an additional 7 min. Helium served as the carrier gas, flowing at a constant rate of 0.80 mL/min. The MS conditions in the study included an EI ion source, an electron energy set at 70 eV, and a mass acquisition range from 33 to 495 *m*/*z*. Through mass spectral comparison with the NIST17 mass spectral collection, the volatile chemicals were identified.

### 2.7. Statistical Analysis

Every experiment was conducted three times, and the significance was determined through statistical analysis using SPSS 26 (SPSS Inc., Chicago, IL, USA). Differences were assessed using Duncan’s test at a significance level of *p* < 0.05. The results are presented as the mean ± standard deviation, and the graphs were created using Origin 2021 (Northampton, MA, USA).

## 3. Results and Discussion

### 3.1. Gelatinization Properties of Starch in Composite Wheat–Bean Flours

The effects of diverse pretreatments on the gelatinization properties of red kidney bean blends were determined using DSC (Table 1). The starch gelatinization absorption peak for wheat flour was observed at 63.56 °C, which is typical for this type of starch and consistent with the reported values [36]. Compared with wheat flour, the onset (To) and peak (Tp) gelatinization temperatures of composite flours were significantly elevated (*p* < 0.05), predominantly due to the relatively higher amylose content in the bean flour, which forms insoluble complexes with lipids during swelling and gelatinization, thereby delaying the gelatinization of the red kidney bean flour blends [37]. Among the various pretreatments, both atmospheric and high-pressure steaming augmented the To and Tp values of composite flours, which was ascribed to the alterations in the internal structure of legume starch granules caused by the hydrothermal treatment, mainly involving the interaction between amylopectin–amylopectin and starch–lipid components [38]. In contrast to wheat flour, the gelatinization enthalpy (ΔH) of composite flours was relatively lower, potentially associated with their lower overall starch content resulting from the replacement of wheat flour with RKBF and gluten flour. In addition, differences in starch composition, including the reported amylose and amylopectin characteristics of kidney bean starch, may also contribute to this behavior [39]. Notably, after thermal treatment, the ΔH value decreased significantly by 4.6% (roasting) to 13.2% (high-pressure steaming) (*p* < 0.05), which could be attributed to partial gelatinization of the red kidney bean starch during the pretreatment process. Particularly, after high-pressure steaming, the red kidney bean premix manifested the lowest ΔH value, possibly related to the reduction in the crystallinity of the starch during the treatment. Similar phenomena have also been reported in other legumes. For example, thermal treatment significantly decreased the ΔH of lentil flour from 4.60 J/g to 1.56 J/g, which was attributed to the partial gelatinization of starch and the associated structural changes during processing [40]. Another study also reported a decrease in the crystallinity index of alfalfa seeds following treatments such as germination and drying, further suggesting that processing can disrupt the ordered crystalline structures of legume materials [41]. Furthermore, the extent of hydrothermal treatment correlated with the degree of starch disruption, leading to a more pronounced decrease in the gelatinization enthalpy. Other pretreatments did not exert a distinct influence on the gelatinization characteristics of the blended powder.

### 3.2. Mixing Properties of Composite Wheat–Bean Flours

Table 2 illustrates a comparison of the mixing properties among different pretreated red kidney bean premixes. The incorporation of RKBF led to a notable enhancement in the water absorption (WA) of dough from 67.8% to 80.3% (*p* < 0.05) owing to the high water-holding ability of bean flour [42]. On the one hand, the elevated dietary fiber content in red kidney bean hulls enhances water absorption through hydrogen bonding interactions between the fibers and water molecules. On the other hand, the proteins found in beans, predominantly globulins, function as storage proteins with a significant capacity for water retention [12]. Compared with untreated RKBD, the heat-treated groups considerably enhanced the water absorption rate by 5.7–9.7% (*p* < 0.05). The results are in line with prior research on heat pretreatment of bean seeds [43,44]; thermal denaturation of proteins and expansion of crude fibers during starch gelatinization and heating might cause an increase in water absorption. The sprouting treatment had no significant impact on the dough’s water absorption, while the peeling treatment led to a decrease due to the loss of high water-absorbing components from the bean hull.

The dough development time (DDT) is correlated with the gluten level, while the stability time (ST) is associated with the structural strength of the gluten protein matrix [45]. The DDT and ST of RKBDs with different pretreatments were lower than those of wheat flour dough (WFD), which could be attributed to the diluting impact of gluten proteins and damaged starch. Due to the slow hydration of gluten proteins, wheat flour with high gluten content takes longer to form a viscoelastic dough [46]. A similar trend was observed, indicating that combining whole wheat flour with flours from various legumes reduced DDT and ST levels when bean flour exceeded 15% [47]. Compared with untreated RKBD, the peeling and sprouting treatments resulted in reductions in DDT by 21.4% and 22.1% (*p* < 0.05), respectively, while other pretreatments did not exhibit a significant impact. Regarding ST, thermal pretreatment significantly enhanced the ST values by 17.6–34.3% (*p* < 0.05), indicating a more robust gluten network and superior resistance to mechanical mixing and processing conditions. This improvement might be attributed to the reduction in proteolytic enzyme activity within the legume flour during the heat treatment process, which consequently enhances the stability of legume dough [48]. The sprouting treatment had no significant effect on ST, whereas peeling resulted in an 18.6% decrease. This phenomenon might be attributed to the high dietary fiber content in bean skin. Research has demonstrated that in breads characterized by low gluten content or gluten-free formulations, suitable dietary fiber exhibits remarkable water-holding capacity and thickening properties [49]. These fibers can establish a three-dimensional network structure within the dough, thereby enhancing its viscoelasticity and stability, which ultimately leads to improvements in structural attributes such as specific volume and texture of the bread. However, some other evidence suggests that dietary fiber may also contribute to the reduction in dough stability. Dietary fiber can compete with gluten proteins for water and physically interfere with the formation of the gluten network, thereby inhibiting gluten development and weakening dough stability [50]. Therefore, the shortened stability time may result from the combined effects of gluten dilution and the interference of dietary fiber with gluten network formation.

The weakening degree (WD) serves as an indicator of the dough’s resistance to mixing. The incorporation of RKBF compromised the gluten network structure to varying extents when compared with WFD. All pretreatments improved the dough’s WD, with roasting and high-pressure steaming demonstrating relatively superior effects. The setback value (SV) indicates the bread’s aging after baking. Adding RKBF significantly reduced the aging value of the dough (*p* < 0.05), likely due to the enhanced water absorption capabilities of the dietary fiber and proteins in the bean flour, which inhibit starch–water interactions and reduce starch retrogradation. After pretreatment, peeling accelerated dough aging, while sprouting treatment had no significant effect. In the heat treatment group, all treatments except roasting showed considerable potential in improving bean dough aging (*p* < 0.05). This result is consistent with previous studies on the incorporation of other legume flours. For example, in a study investigating the thermomechanical properties of wheat flour substituted with 5%, 10%, and 15% chickpea, broad bean, common bean, and red lentil flours, higher proportions of legume flour were found to enhance the resistance of the system to starch retrogradation, with the extent of the effect closely related to the type and incorporation level of the legume flour [51]. Notably, the relationship between the incorporation level of legume flour and bread staling does not necessarily exhibit a simple linear trend. In the study comparing 5% and 10% non-germinated and germinated alfalfa seed flours, both the incorporation level and germination treatment were shown to alter the thermomechanical properties of wheat dough [52]. On the other hand, a study on roasted chickpea flour showed that an incorporation level of 10% had only a minor effect on dough and bread quality, whereas increasing the incorporation level to 15–20% instead promoted bread staling [17]. These results indicate that the effect of legume flour on SV depends not only on the type of legume but also on the incorporation level.

### 3.3. Fundamental Rheological Parameters of Composite Wheat–Bean Doughs

**Figure 1:**
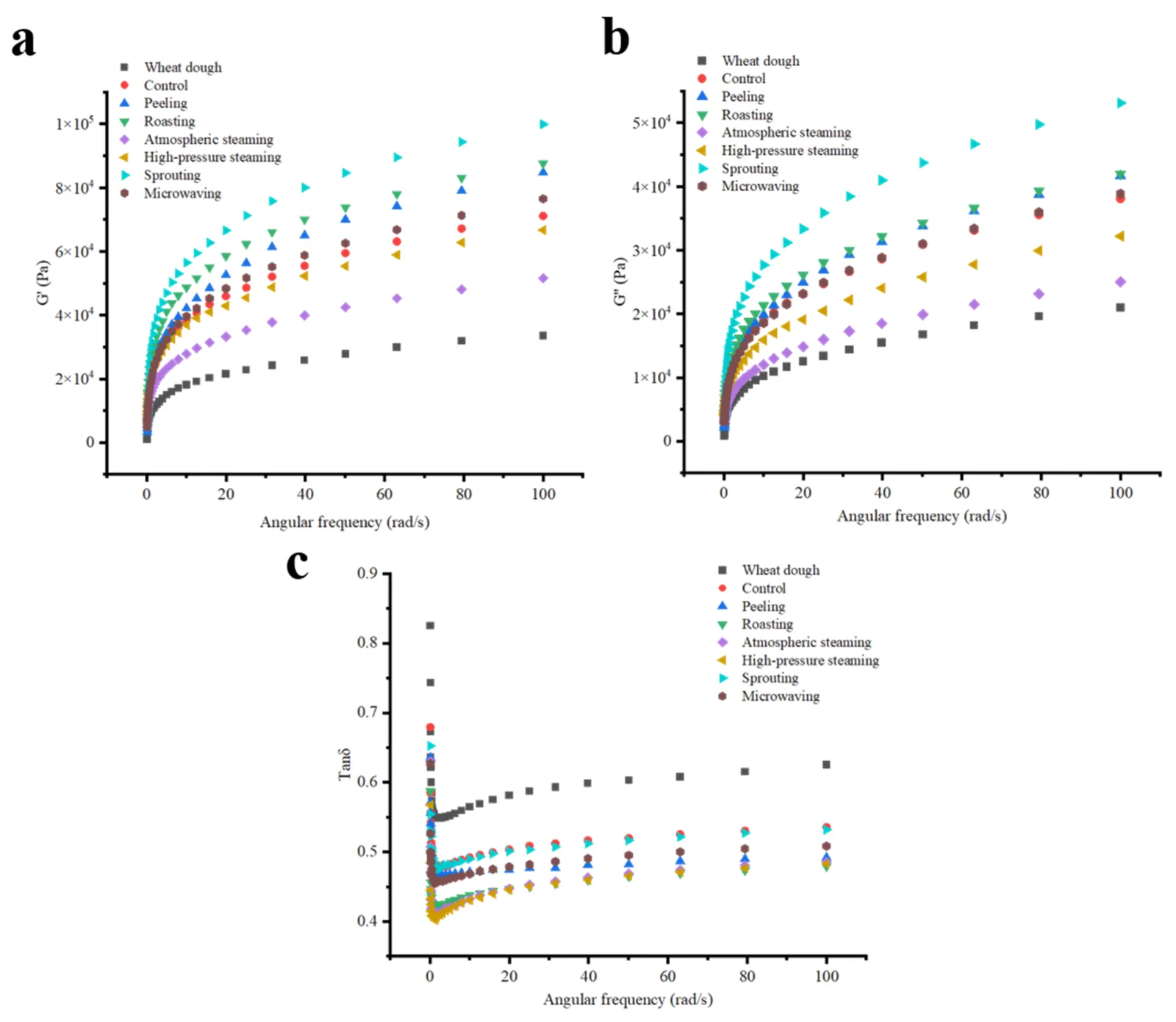
Effect of different pretreatments on the (**a**) storage modulus (G′), (**b**) loss modulus (G″), and (**c**) loss tangent (tan δ) of red kidney bean doughs (RKBDs).

Frequency scanning is a method used to determine the dynamic rheological properties of dough, providing parameters for energy storage modulus (G′), loss modulus (G″), and loss angle tangent (tan δ), which indicate the interaction strength and stability of dough [53]. The frequency scanning results are shown in Figure 1. It was observed that the G′ and G″ of all dough samples increased with frequency and G′ > G″, suggesting that samples of all groups exhibited weak gel properties and were dominated by solid-like behavior within the frequency range of 0.1~100 rad/s.

At the same frequency, both G′ and G″ of the RKBD were significantly higher than those of the WFD (*p* < 0.05), which might be due to the protein and dietary fiber from red kidney beans hindering gluten protein hydration or inhibiting gluten network development, thereby altering dough performance [18]. Among pretreated groups, the G′ and G″ after sprouting treatment were the largest, followed by roasting. The smallest values were found in dough treated with atmospheric steaming, and high-pressure steaming ranked second; both were lower than the untreated control dough. Research has demonstrated that the increase in G′ and G″ values within legume–wheat composite dough usually signifies enhanced viscosity and elasticity, consequently leading to the formation of a more robust dough network structure, which often results in suboptimal baking performance [17,54]. Both roasting and sprouting treatments significantly enhanced the viscoelastic index of the dough (*p* < 0.05). During roasting, increased protein denaturation intensifies competition for water-binding sites within the dough, consequently disrupting the continuous network established by starch and proteins [55]. In contrast, sprouting activates endogenous hydrolytic enzymes, leading to protein and starch degradation and redistribution of water among dough components. These compositional and structural modifications may interfere with the development of the gluten network and viscoelastic behavior of the composite dough [56]. Relevant findings have also been reported, showing that the incorporation of 30% sprouted and roasted pea flour into wheat flour increased the G′ and G″ values of the dough, as well as its hardness following both pretreatments [57]. In contrast, atmospheric and high-pressure steaming have the capacity to improve the viscoelastic properties of RKBD. During the hydrothermal treatment process, a portion of the starch contained in bean flour undergoes pregelatinization while concurrently facilitating protein denaturation and fiber modification. These alterations reduce the interactions between these components and water, thereby promoting their effective integration into the dough matrix and contributing to the formation of a softer dough with improved processing characteristics [26].

The tan δ indicates the ratio between G″ and G′. All samples exhibited a decreasing and then increasing trend in tan δ, indicating that the dough displayed more solid-like characteristics under low-frequency shear and more liquid-like properties under high-frequency shear. Incorporating bean flour decreased the tan δ of the doughs, suggesting that adding RKBF increased polymer content and transformed the gluten network structure into a more elastic and rigid form [58].

### 3.4. FTIR Spectroscopy Analysis of Protein in Composite Wheat–Bean Doughs

The protein network structure of gluten is intimately related to the secondary structure and composition of dough gluten proteins, directly impacting bread quality. In the composite bread, the incorporation of high-content RKBF leads to a pronounced alteration in the Fourier Transform Infrared (FTIR) spectrum of the dough sample, predominantly reflected in the protein-related region of the amide I band spanning 1600 to 1700 cm^−1^. Within this specific wavenumber range, the principal secondary structures of proteins are characterized by α-helix (1650–1660 cm^−1^), β-sheet (1610–1640 cm^−1^), β-turn (1660–1700 cm^−1^), and irregular curling (1640–1650 cm^−1^) [59].

As shown in Table 3, in contrast to WB, the β-sheet content in RKBDs exhibited a remarkable increase (*p* < 0.05), while, correspondingly, the α-helix, β-turn, and irregular curling structures decreased. Comparable phenomena have also been documented in bread produced from composite flours of wheat flour and other legumes [12,17,18]. This phenomenon is partly due to the inherently high β-sheet structure of globulins in red kidney beans [60]. In addition, when a large amount of RKBF is mixed with wheat flour, the competition between fiber and protein for water leads to dehydration and condensation of gluten proteins, causing a shift to the β-sheet structure, and this structural change is often related to poor bread quality [61]. In this experiment, the untreated group had a β-sheet content of 36.35%, significantly higher than the 31.12% observed in WFD (*p* < 0.05). Following pretreatments, the atmospheric and high-pressure steaming groups demonstrated reductions to 33.39% and 32.94%, respectively, while other groups showed less pronounced improvements. A higher proportion of α-helix typically indicates a more organized protein secondary structure, which improves dough hardness [62]. In this paper, atmospheric (18.79%) and high-pressure steaming (18.86%) treatments both increased the α-helix content compared to the untreated group (17.42%) (*p* < 0.05), while no significant change was noted in other pretreatment groups. This phenomenon might be attributed to the disruption of the initial hydrogen bond system caused by water migration and heat transfer during atmospheric and high-pressure steaming, leading to the formation of fresh noncovalent crosslinks within or between protein molecules, thereby altering their secondary structure [63]. Other studies have also demonstrated that treating cereal flour with saturated steam leads to a more ordered secondary structure in the proteins [64].

### 3.5. GMP and SHf Content of Composite Wheat–Bean Doughs

**Figure 2:**
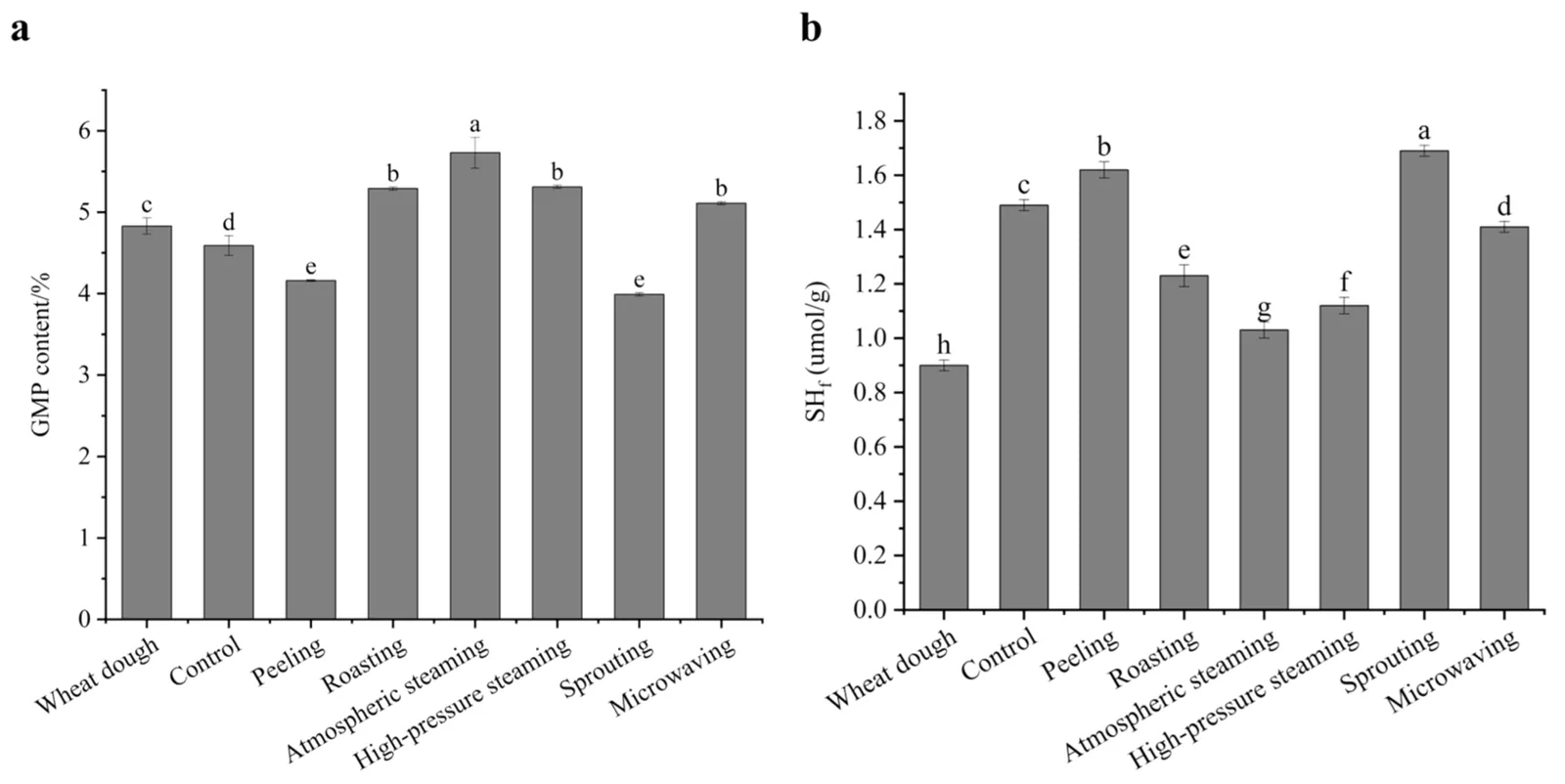
Effect of different pretreatments on the (**a**) glutenin macropolymer (GMP) and (**b**) free sulfhydryl groups (SHf) content of RKBDs. Different letters in the same figure indicate significant differences at the 0.05 level (*p* < 0.05).

Gluten macropolymer (GMP) pertains to the portion of gluten that cannot be extracted by SDS solution, which has a high proportion of HMW-gluten subunits and contributes significantly to dough strength and baking quality [65]. As depicted in Figure 2a, diverse pretreatments exert varying influences on the GMP content in dough. The significantly lower GMP content in untreated RKBD (4.59%) compared with WFD (4.83%) can primarily be attributed to the dilution effect caused by the high proportion of bean flour (*p* < 0.05), which hindered GMP polymerization and increased its solubility. Additionally, moisture competition and the physical barrier created by dietary fiber impeded gluten protein formation, further reducing GMP content [66]. In contrast, after heat treatment, the GMP content in RKBDs significantly increased to 5.11% (microwaving)–5.73% (atmospheric steaming) (*p* < 0.05). This increase is mainly due to heat-induced alterations in the structure of original proteins in bean flour, leading to decreased solubility and consequently higher GMP production. Previous studies have also shown that inactive soy flour increased the GMP content in dough mixtures, whereas active soy flour had the opposite effect [67], which is consistent with the trend observed in the peeling (4.66%) and sprouting (3.99%) treatments. This difference indicates that the unfolded denatured bean protein exhibits a higher degree of interaction with wheat protein, potentially enabling it to substitute for a portion of gluten protein in forming GMP. However, despite the total protein content of GMP being higher than that of wheat samples, the interaction between wheat and bean proteins appears to be diminished.

The free sulfhydryl group (SHf) content of various pretreatment groups is illustrated in Figure 2b. Wheat dough exhibited the lowest SHf content at 0.9 μmol/g, and the incorporation of RKBF elevated the SHf content to a range of 1.03–1.69 μmol/g (*p* < 0.05). The change in SHf content can be used to explain the process of GMP formation: when the free sulfhydryl groups are oxidized to disulfide bonds, this promotes the polymerization of GMP, thus increasing its content [68]. Among the pretreated groups, the heat-treated group demonstrated a lower SHf content (1.03–1.41 μmol/g) compared with the untreated group (1.49 μmol/g) (*p* < 0.05), which corresponded to the higher GMP content. Notably, the SHf content was particularly low in both the atmospheric steaming (1.03 μmol/g) and the high-pressure steaming (1.12 μmol/g) groups. The reduced SHf content in dough containing hydrothermally treated RKBF may reflect enhanced oxidation and protein–protein association during dough mixing [69]. Heat-induced structural changes in kidney bean proteins may expose reactive groups and promote interactions with wheat gluten proteins. However, the present results do not directly demonstrate the formation of intermolecular disulfide bonds between kidney bean and wheat proteins. The decrease in SHf content reflects more extensive covalent cross-linking between proteins, which in turn enhances the structural strength of the gluten network.

### 3.6. Microstructure Analysis of Composite Wheat–Bean Doughs

Confocal laser microscopy (CLSM) can be employed to visually understand the impact of differently pretreated RKBF on the gluten network structure. The non-covalently labeled gluten protein with rhodamine B is shown in red, while the non-covalently labeled pentosan and starch granules appear in green, and the yellow part emerges when the two channels overlap, signifying that starch interacts with or is encased in the gluten protein [70].

**Figure 3:**
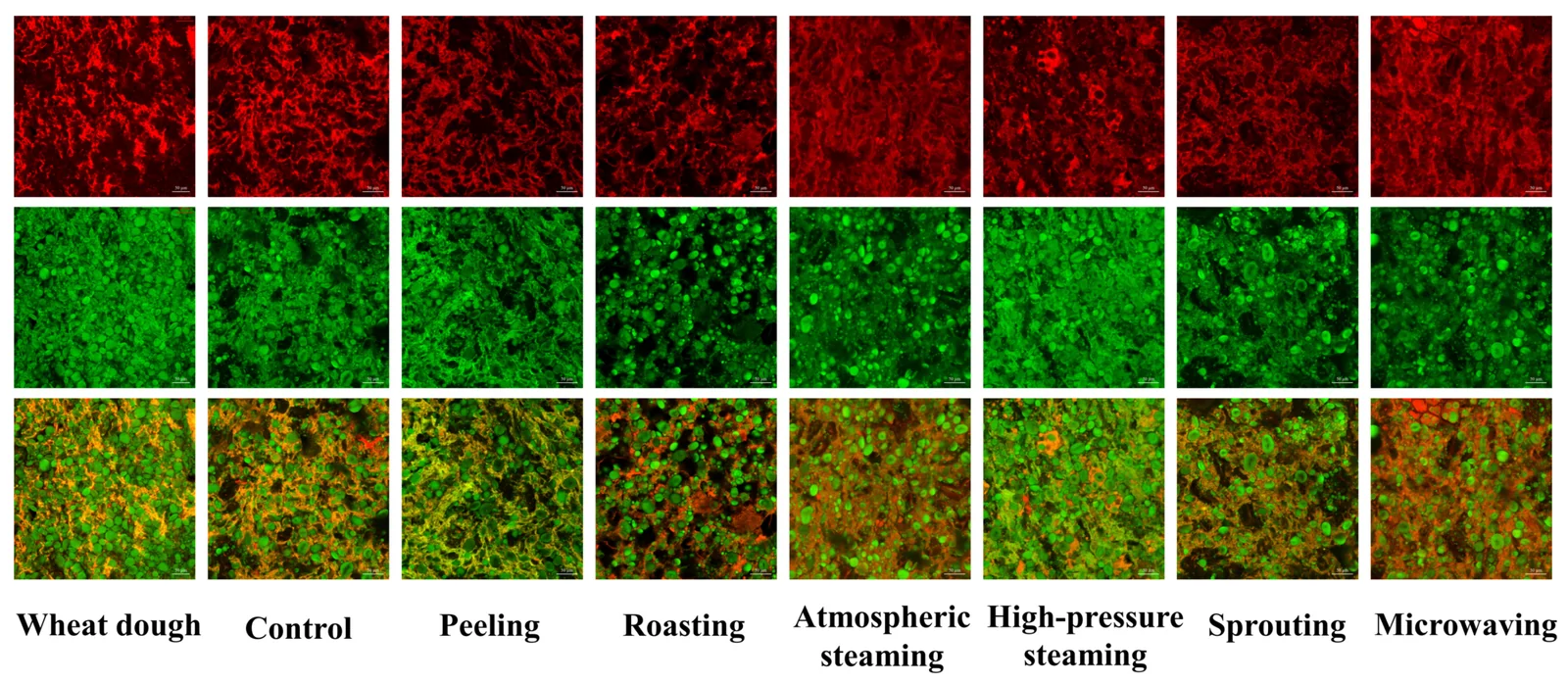
Effect of different pretreatments on the microstructure of red kidney bean doughs (RKBDs). Red: gluten proteins stained with Rhodamine B; Green: pentosans and starch granules stained with FITC; Merged: yellow regions indicate colocalization, suggesting interaction or encapsulation between starch and gluten.

Figure 3 shows the WFD’s protein matrix encircled by a starch network, with smaller protein molecules integrated within it, and the continuous gluten network structure identifiable. Conversely, adding RKBF reduced the gluten protein level and impeded gluten network formation. As a result, the gluten network of the control group appeared loose and uneven, resembling a sheet with visible breaks, and many starch granules were visible or even dissolved. Among the experimental groups, the starch distribution of the RKBD was relatively more homogeneous after atmospheric and high-pressure steaming treatments. Nevertheless, there was a reduction in both the quantity of large starch granules and the degree of starch aggregation. This could be attributed to the destruction of the branched starch structure during heat treatment, leading to an increase in the straight-chain starch proportion and a decrease in the degree of starch aggregation [71]. This phenomenon was more pronounced after roasting, with the dough exhibiting a significant number of irregular structural voids. The addition of sprouted RKBF also reduced the starch–protein content ratio in the bread by introducing protein and starch hydrolases, impairing the technical properties of starch and gluten proteins in the bread system [48]. Similar microstructural changes have also been reported in wheat dough enriched with germinated bean flour. As the incorporation level of germinated bean flour increased, the starch regions in the dough decreased markedly. Meanwhile, the introduction of non-gluten proteins, dietary fiber, and active enzymes from bean flour induced a gluten dilution effect and altered the gluten network structure, thereby affecting the viscoelastic properties of the dough, which is generally consistent with the present study [72]. However, the effects of different legume flours on dough microstructure are not entirely consistent. For example, although the addition of germinated lentil flour similarly increased the protein regions and decreased the starch regions, the dough matrix remained relatively intact even at higher incorporation levels [73]. These differences suggest that the effects of legume flour on wheat dough microstructure may be related not only to gluten dilution but also to the type and incorporation level of legume flour, as well as changes in dough components during germination.

### 3.7. Baking Performance and Quality Attributes of Composite Bean-Enriched Bread

**Figure 4:**
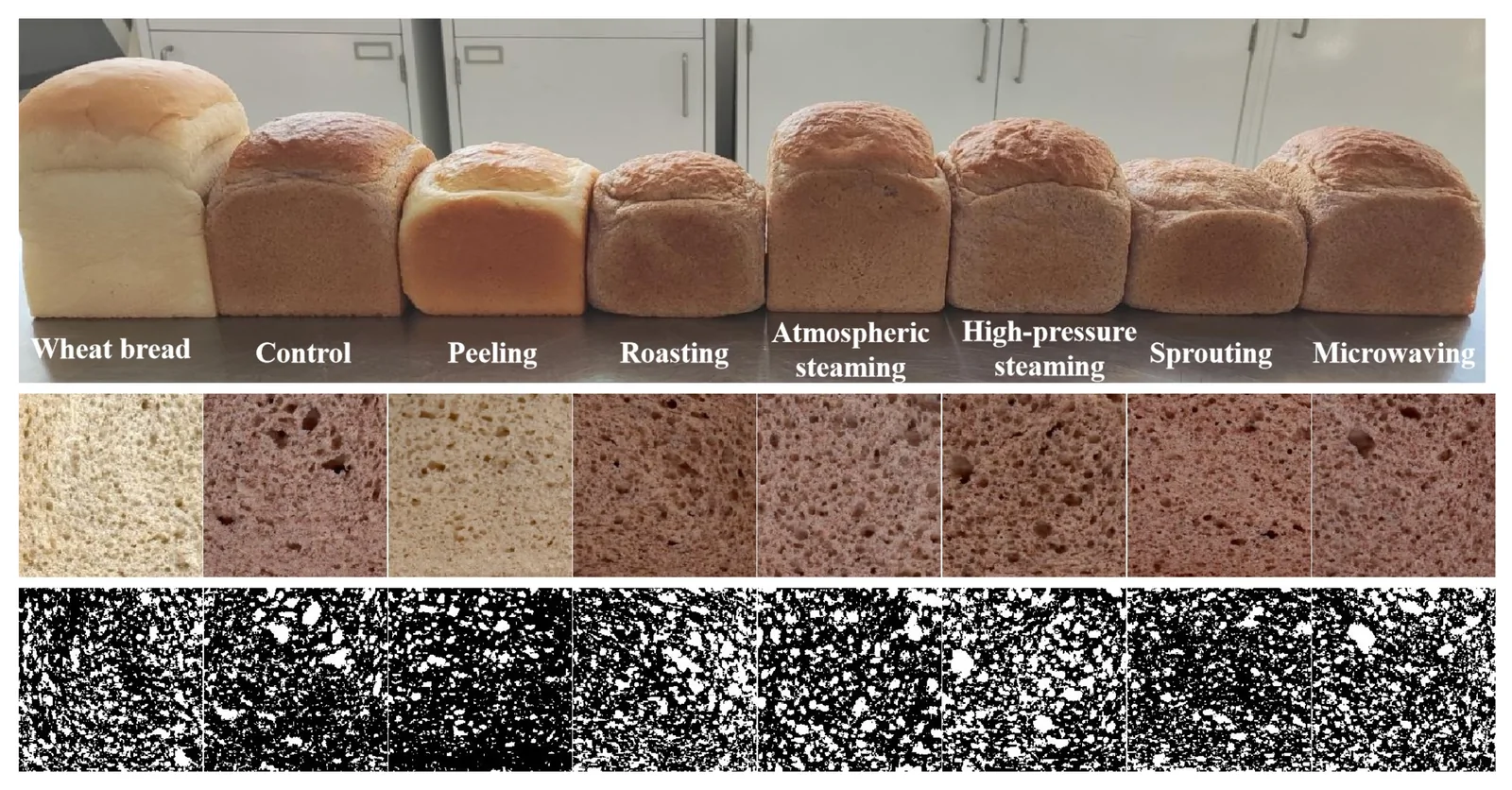
Effect of different pretreatments on the appearance, morphology, and pore structure of red kidney bean bread (RKBB).

Figure 4 illustrates the morphological appearance of different preprocessed RKBB, along with two-dimensional plane scanning images from the scanner and cross-sectional analysis images processed by ImageJ software. Compared with WB, RKBB exhibited significant volume reduction ranging from 20.67% (atmospheric steaming) to 46.07% (roasting) (*p* < 0.05) due to gluten dilution, which led to impaired dough structure and reduced gas retention capacity (Table 4) [71]. Among all pretreatment groups, only atmospheric steaming significantly enhanced the specific volume of RKBB from 3.37 cm^3^/g to 3.53 cm^3^/g (*p* < 0.05), while simultaneously optimizing its porous structure and improving overall uniformity. Despite legumes having a relatively low content of sulfur-containing amino acids, hydrothermal treatment has been shown to facilitate protein aggregation and covalent cross-linking [69], thereby strengthening dough structure and increasing bread volume. Comparable findings were reported regarding the effects of heat treatment of sorghum flour on the quality and functional properties of gluten-free bread [74]. In contrast, the high-pressure steaming group did not exhibit significant changes; however, substantial deterioration in bread quality was observed following peeling, roasting, and sprouting treatments. This decline might be attributed to both the dilution of the gluten structure and adverse effects stemming from protein denaturation induced by heat treatment, as well as enzymatic degradation.

The texture findings of the bread conformed to the patterns observed in its rheological and specific volume properties. Adding RKBF significantly increased bread hardness and chewiness (*p* < 0.05) while decreasing springiness, resilience, and cohesiveness, leading to a deterioration in bread quality. With respect to hardness, only the atmospheric steaming group demonstrated a positive effect, resulting in a 16% reduction compared with the untreated RKBB. In terms of springiness, all heat treatment groups, excluding the microwaving treatment, exhibited effective improvements ranging from 3% to 5%. Regarding cohesiveness, all methods except for the sprouting treatment produced increases between 5% and 25%. Similarly, resilience showed an enhancement ranging from 4% to 39%, again excluding the sprouting treatment. These results were generally consistent with the findings reported for wheat breads enriched with other legume flours. For example, the incorporation of 30% faba bean flour into wheat bread decreased the specific volume while increasing bread density and hardness. However, low-intensity pulsed electric field treatment was also applied in that study, which may have affected the observed results [75]. A recent study on lupin-enriched wheat bread also showed that replacing wheat flour with 15–30% lupin flour significantly altered bread hardness, springiness, cohesiveness, and resilience. Direct incorporation of lupin flour generally increased bread hardness, whereas the incorporation of fermented lupin flour as sourdough at a 15% substitution level alleviated the increase in hardness to some extent [76]. These findings suggest that the incorporation of legume flour has adverse effects on the texture of wheat bread, particularly by increasing hardness and reducing elasticity-related properties.

The color of the bread has a significant influence on customers’ purchasing decisions. Compared with WB, adding RKBF decreased the L* and b* values of the bread crust (except for peeling) and increased the a* values due to an elevation in protein content from bean flour, intensifying Maillard and caramelization reactions on the bread surface [77]. For bread crumb, the reduction in L* values and increase in a* and b* values for all pretreated groups were greatly influenced by the color of the RKBF itself, with peeled RKBB being the closest in color to WB. The rise in the a* and b* values of crumb color indicated dilution of the white wheat endosperm by the darker RKBF, thus increasing red and yellow undertones.

The whiteness index (WI), browning index (BI), and total color difference (ΔE) further confirmed the effects of RKBF incorporation and different pretreatments on the overall color characteristics of the bread. Compared with WB, the WI of the RKBB crust decreased from 50.64 to 33.88–43.23, whereas the BI showed an increasing trend, indicating that RKBF incorporation reduced crust whiteness and promoted browning. Meanwhile, the ΔE values of the RKBB crust ranged from 10.12 to 25.55, with the peeling group exhibiting the lowest ΔE, suggesting that peeling could mitigate the crust color changes caused by RKBF incorporation. A similar trend was observed in the crumb. The WI decreased from 65.27 in WB to 45.82 in the high-pressure steaming group, while the BI increased from 15.88 to 39.84 in the roasting group, and the ΔE values ranged from 5.4 to 19.93.

The color changes have also been reported in other legume-enriched wheat breads. For example, as the incorporation level of germinated chickpea flour increased from 5% to 20%, the L* values of both the crust and crumb gradually decreased, whereas the a* and b* values generally increased, indicating that legume flour incorporation resulted in a darker bread with more pronounced red and yellow tones [78]. Similar decreases in L* and increases in a* and b* have also been observed in wheat bread enriched with germinated lentil flour, and these changes were attributed not only to the pigments and phenolic compounds inherent in the legume flour but also to the increased protein and amino acid contents introduced by the flour, which may promote Maillard reactions during baking [73]. These trends are generally consistent with those observed for the crumb of RKBB in the present study. However, the b* value of the RKBB crust showed a decreasing trend, indicating that the color responses may vary depending on the type of legume material and the bread region analyzed. This difference may be related to the intrinsic pigment composition of red kidney beans. In addition, a recent study on red lentil-enriched wheat bread demonstrated that both legume incorporation and pretreatment could significantly affect the overall color of the bread crust and crumb through changes in BI and ΔE [79]. Therefore, the results of this study are generally consistent with the color changes reported for other legume-enriched wheat breads, while the differences among pretreatment groups further indicate that RKBF pretreatment is also an important factor regulating the final bread color.

### 3.8. Protein Content and Amino Acid Composition of Composite Bean-Enriched Bread

RKBB exhibited a significantly higher protein level (29.43–30.96%) compared with WB (16.85%) (*p* < 0.05) due to the intrinsically higher protein content of the raw material. Among pretreated groups, peeled RKBB manifested a marginally higher protein content. According to both the FDA (requiring ≥10 g protein per RACC, i.e., ≥20% of the 50 g DV) and China’s GB 28050-2011 (requiring ≥20% of the energy value for solid foods) [80], a food meeting either criterion may be described as “high protein.” All RKBB in this experiment evidently satisfied these requirements.

The quantity and proportion of amino acids within proteins determine their nutritional value. The distribution of amino acid fractions was thoroughly investigated by measuring 18 amino acids in each sample, including eight EAAs. Although wheat is abundant in sulfur-containing amino acids, including methionine and cysteine, it has comparatively low levels of lysine. In contrast, red kidney beans demonstrate a complementary profile of these EAAs. The inclusion of RKBF boosted the bread’s lysine content from 19.44 mg/g to 30.73–32.71 mg/g (*p* < 0.05), while the sulfur-containing amino acid content of RKBB was also comparable to that of WB. This is consistent with previous findings showing that the incorporation of raw and germinated ayocote bean flour into wheat flour for nutritional fortification not only improved the amino acid composition of proteins but also increased the content of essential amino acids, particularly lysine, the limiting amino acid [81]. Similar improvements in amino acid composition have also been reported in other legume-enriched wheat breads. A recent study on wheat breads enriched with cowpea and common bean flours showed that lysine was the major limiting amino acid in wheat bread, whereas the incorporation of legume flours increased the lysine content, with a more pronounced improvement observed at higher substitution levels [82]. The amino acid profile of the bread analyzed in this study was compared to the standard amino acid pattern recommended by the Food and Agriculture Organization of the United Nations (FAO) [83], as illustrated in Table 5. Notably, RKBB demonstrated higher levels of nearly all amino acids, while its sulfur-containing amino acid content was similar to that of WB, resulting in a more balanced protein amino acid composition aligned with FAO recommendations. Additionally, it was found that the EAA/TAA of RKBB was 11.93–13.83% higher than that of WB (*p* < 0.05), and pretreatment did not exert a significant influence on this ratio.

Egg protein is widely recognized as an excellent source of high-quality protein, featuring a balanced and comprehensive amino acid profile, and the EAAI is commonly used to evaluate the quality of different protein sources by measuring how closely the amino acid profiles of experimental proteins match that of egg protein. In this study, the EAAI value of RKBB (45.75–50.26%) was significantly greater than that of WB (43.68%) (*p* < 0.05). Additionally, the analysis of samples subjected to various pretreatment methods revealed that the EAAI value for the atmospheric steaming group (50.26%) did not significantly differ from that of the untreated control group (50.02%), with both groups showing significantly higher EAAI values than WB (*p* < 0.05). This indicated that atmospheric steaming did not negatively affect the amino acid composition, thus preserving its nutritional value. However, other pretreatment groups had slightly lower EAAI values than the untreated group, likely due to degradation or transformation of some amino acids during pretreatments, resulting in amino acid loss and affecting overall protein quality.

### 3.9. In Vitro Starch Digestibility and Glycemic Potential of Composite Bean-Enriched Bread

**Figure 5:**
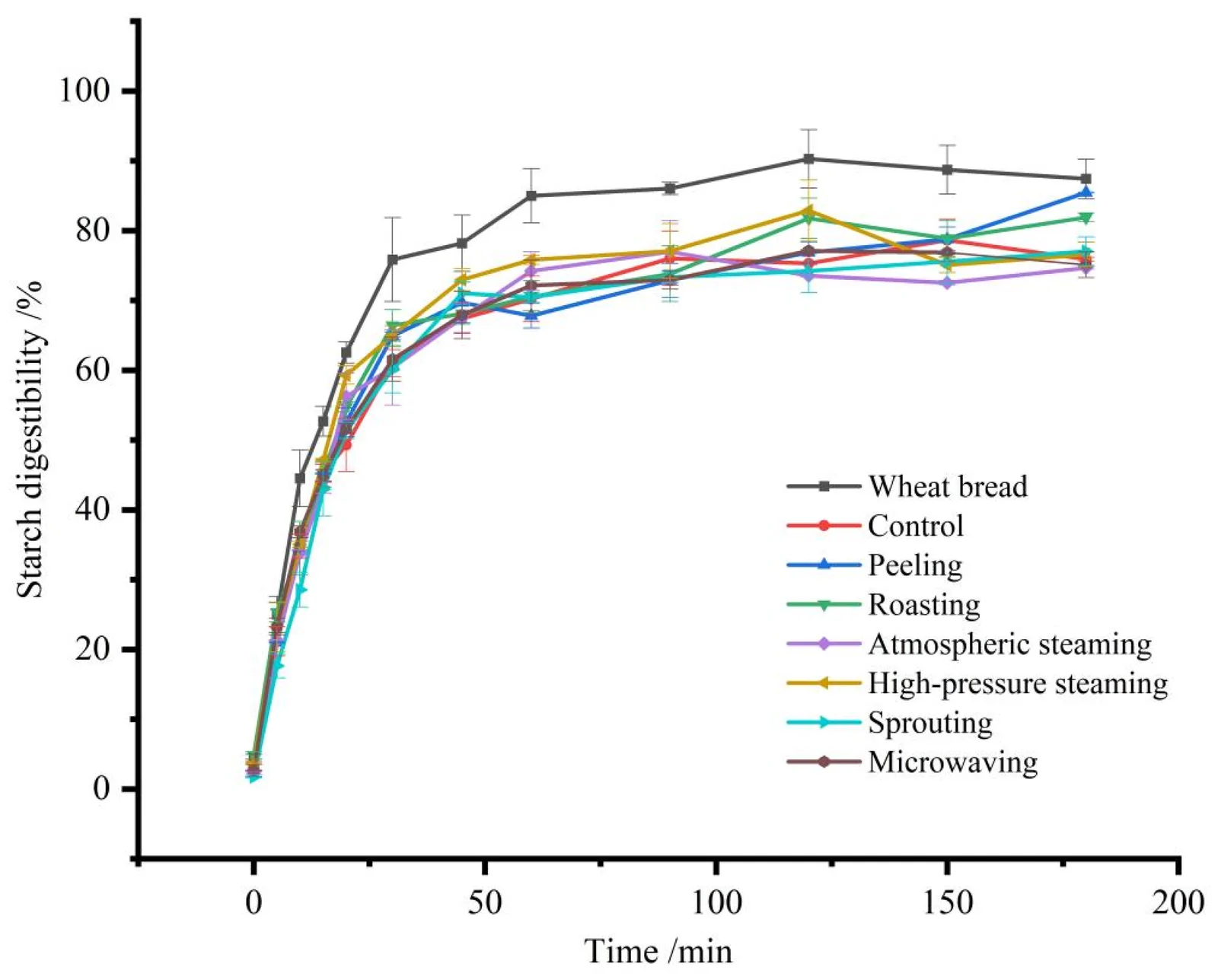
Effect of different pretreatments on the in vitro digestive curve of red kidney bean bread (RKBB).

Amid the escalating emphasis on healthy dietary choices, bread featuring low glucose release holds promising market potential. The impact of different pretreatments of RKBF on the in vitro digestion of starch in bread samples was investigated using in vitro methods, as shown in Figure 5 and Table 6. The addition of RKBF significantly reduced the total starch content from 59.97% in WB to 40.58–43.25% in RKBB (*p* < 0.05). Overall, all bread samples displayed a similar digestion curve, with a sharp increase in starch hydrolysis rates during the first 30 min of intestinal digestion and a gradual return to equilibrium after 120 min.

Kinetic analysis revealed that the addition of RKBF lowered the final concentration and starch hydrolysis rate at equilibrium. Additionally, adding RKBF significantly reduced the kinetic constant K (*p* < 0.05), with the exception of the atmospheric and high-pressure steaming treatments. The eGI values were lower than those of WB in all RKBB groups, potentially due to the dietary fiber in RKBF binding to protein and forming a barrier around starch particles, which hinders enzymatic hydrolysis [84]. The eGI values of the peeling, roasting, and high-pressure steaming groups exhibited a significant increase compared with the untreated group (*p* < 0.05), presumably because these processes promoted the exposure of starch to digestive enzymes.

RDS is quickly absorbed and digested within the gastrointestinal tract, leading to a rapid elevation in blood glucose levels. In contrast, SDS is digested slowly and fully releases glucose over a long period, while RS remains undigested in the small intestine [85]. Compared with the WB, there was a significant decrease in the RDS of RKBB from 64.91% to 48.99–56.16% and an increase in RS from 11.76% to 21.27–25.55% (*p* < 0.05). The inclusion of RKBF also led to a significant reduction in the SDS content of the bread (except for sprouting) (*p* < 0.05), consistent with the observed trend in eGI values. Among the pretreatment groups, RKBB treated with atmospheric steaming presented the lowest eGI value, which was reflected in lower RDS and SDS contents and higher RS content. This reduction could be attributed to the enhanced migration of starch chains produced by hydrothermal treatment, which reduces enzyme sensitivity [86]. Similar results have also been observed for buckwheat starch modified by heat–moisture treatment [87]. Following treatment, the RDS content decreased significantly, while the SDS and RS contents increased due to enhanced interactions between amylose–amylose and amylose–amylopectin post-processing, leading to tougher and less digestible starch crystals.

### 3.10. Volatile Compounds of Composite Bean-Enriched Bread

**Figure 6:**
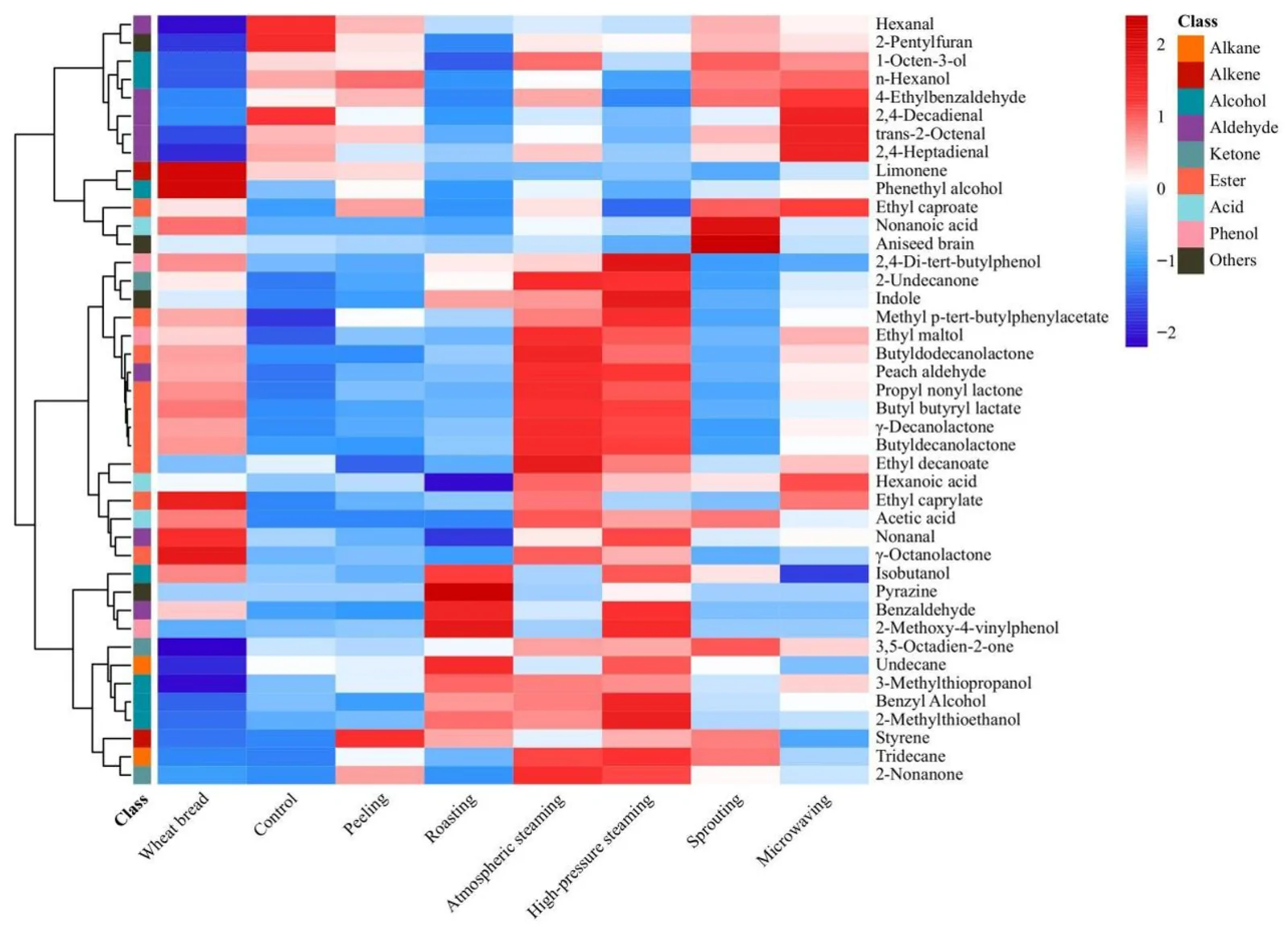
Effect of different pretreatments on the volatile components of red kidney bean bread (RKBB).

In this work, a hierarchical cluster analysis (HCA) method was employed to investigate the variations in the concentration of volatile compounds in bread crumbs after different pretreatments. The analysis results are presented visually through a heat map with dendrograms. Figure 6 illustrates how the addition of RKBF affects the main volatile flavor components in the bread crumbs. A total of 42 volatile flavor compounds were identified, including two alkanes, seven alcohols, eight aldehydes, three ketones, three acids, ten esters, three phenols, and four heterocyclic compounds.

The higher alkanes (undecane and tridecane) found in RKBB were primarily derived from the oxidative breakdown of lipids, indicating the start of lipid oxidation, which produces the characteristic aroma of beans [88]. Most of the seven alcohols identified were also products of lipid oxidation, and the relative contents of 2-phenylethanol and n-hexanol were high in RKBB. According to previous studies, 2-phenylethanol has an odor of honey and yeast and is an important flavor-active ingredient in bread crumbs [89]. The relative content of phenylethanol in WB was 15.35%, which decreased to 3.21% (roasting) and 7.58% (microwaving) with the addition of RKBF. N-hexanol has a typical green and grassy flavor [90], and the addition of RKBF increased its content from 4.18% to 19.51%. Among several pretreatments, heat treatment reduced it to 7.11–15.74%, whereas peeling, sprouting, and microwaving treatments relatively increased its content.

Aldehydes possess a low odor threshold yet contribute significantly to flavor. Hexanal, the major aldehyde in red kidney bean bread crumbs, has a grassy flavor. It is the main odor-presenting component of bean odor, formed by the direct cleavage of lipid hydroperoxides, and has a negative correlation with the scent of WB [91]. The inclusion of bean flour boosted the relative content of hexanal in bread by 130–250%, and heat treatments were the most effective pretreatment methods for red kidney beans, with reductions of 34%, 31%, and 33% after roasting, atmospheric, and high-pressure steaming, respectively. (E,E)-2,4-decadienal originates from the oxidation of linoleate and is also considered an important flavor compound in bread [92]. However, it negatively affects the acceptability of bread flavor [90]; with the addition of RKBF, the (E,E)-2,4-decadienal content increased from 0.60% to 3.43% in WB, and pretreatments reduced it by 43.44–79.30%, except for microwaving (3.79%).

According to Oomah et al. [88], 3,5-octandiene-2-one is also a characteristic volatile compound of kidney beans, primarily derived from amino acid degradation, and has an earthy and green pepper flavor. In this experiment, 3,5-octandiene-2-one was detected in all RKBB groups, though there were slight differences between the groups. 2-amylfuran is generated from amino acid degradation and lipid oxidation, with aromas of bean, green, and soil [93]. Compared with WB, the content of 2-amylfuran in RKBB was increased by twofold. After pretreatments, it decreased by 19.55% (sprouting)–55.10% (roasting).

Pyrazines are one of the most significant volatile compounds generated by the Maillard reaction, and they contribute significantly to bread aroma [91]. Generally, the Maillard reaction in bread occurs mainly in the crust and relatively less in the crumbs. However, pyrazine compounds were still detected in the crumbs of the roasting and high-pressure steaming groups, probably due to the prior occurrence of the Maillard reaction in the bean flour during high-temperature and high-pressure pretreatments. In the roasting-treated group, the relative content of pyrazine compounds had already reached 30%, significantly diluting the undesirable flavors from the beans.

Overall, heat treatment was more effective than other pretreatment methods in reducing levels of “n-hexyl alcohol”, “hexal”, “(E,E)-2,4-sebacedienal”, “3,5-octanediene-2-one” and other beany flavor substances in RKBB, thereby improving the flavor profile of legume bread. It should be noted that the bread formulations contained 15% butter. Therefore, lipids and other components derived from butter may also contribute to the volatile profile of both WB and RKBB, particularly lipid-derived aldehydes, alcohols, and ketones. Accordingly, differences in volatile compounds among samples cannot be attributed exclusively to RKBF.

## 4. Conclusions

In conclusion, incorporating 50% red kidney bean flour into bread significantly enhanced its nutritional profile by boosting protein content from 16.85% to 29.43% and the essential amino acid index (EAAI) from 43.68% to 50.02%, while reducing total starch from 59.97% to 40.91%. However, this high inclusion rate introduced several technical challenges, including increased gelatinization temperature, reduced dough stability, disrupted gluten structure, decreased loaf volume, and undesirable sensory attributes such as hardness and an unpleasant odor. Among the pretreatments investigated, hydrothermal treatments, particularly atmospheric steaming, proved effective in mitigating these issues by reducing the enthalpy of pasting and the free sulfhydryl content, enhancing dough water absorption, processing stability, rheological properties, GMP content, and structural integrity, while also mitigating undesirable bean flavors and preserving amino acids, as well as increasing resistant starch content. Among non-thermal treatments, sprouting treatment increased enzyme activity, leading to protein degradation during mixing and proofing, which affected dough formation time and protein chain conformation. Interestingly, while the peeling treatment mitigated the dark brown color caused by red kidney beans, it exacerbated the deterioration of product volume, a phenomenon that requires further investigation.

This study indicates that adding 50% red kidney bean flour to bread under appropriate pretreatment conditions is feasible. Despite the potential benefits, further optimization of processing parameters and exploration of additives like emulsifiers, enzymes, and hydrocolloids are necessary to fully address the encountered technical difficulties and achieve or surpass the quality standards of traditional wheat bread. Overall, with appropriate pretreatment and process adjustments, the production of high-content red kidney bean bread presents a promising avenue for developing nutritionally enriched bakery products.

## Figures and Tables

**Table 1:** Effect of different pretreatments on the gelatinization properties of red kidney bean doughs (RKBDs).

Samples	Wheat Dough	Control	Peeling	Roasting	Atmospheric Steaming	High-Pressure Steaming	Sprouting	Microwaving
To (°C)	57.63 ± 0.09 ^g^	59.92 ± 0.25 ^cd^	59.21 ± 0.04 ^f^	59.97 ± 0.08 ^bc^	60.44 ± 0.21 ^a^	60.26 ± 0.04 ^ab^	59.64 ± 0.17 ^de^	59.51 ± 0.12 ^e^
Tp (°C)	63.56 ± 0.11 ^d^	65.65 ± 0.19 ^c^	65.61 ± 0.16 ^c^	65.68 ± 0.38 ^c^	66.73 ± 0.27 ^a^	66.22 ± 0.08 ^b^	65.63 ± 0.16 ^c^	65.81 ± 0.03 ^c^
Tc (°C)	69.82 ± 0.48 ^c^	73.09 ± 0.10 ^a^	73.14 ± 0.11 ^a^	72.26 ± 0.23 ^b^	73.38 ± 0.17 ^a^	72.31 ± 0.13 ^b^	73.45 ± 0.14 ^a^	72.31 ± 0.23 ^b^
ΔH_gel_ (J/g)	6.14 ± 0.03 ^a^	3.03 ± 0.07 ^b^	3.09 ± 0.03 ^b^	2.89 ± 0.05 ^c^	2.84 ± 0.01 ^c^	2.63 ± 0.03 ^d^	3.04 ± 0.02 ^b^	2.88 ± 0.02 ^c^

All values are mean ± SD, *n* = 3. Different letters in the same row indicate significant differences between parameters at the 0.05 level (*p* < 0.05).

**Table 2:** Effect of different pretreatments on the mixing properties of red kidney bean doughs (RKBDs).

Samples	Wheat Dough	Control	Peeling	Roasting	Atmospheric Steaming	High-Pressure Steaming	Sprouting	Microwaving
WA (%)	67.8 ± 0.3 ^f^	80.3 ± 0.5 ^d^	75.2 ± 0.3 ^e^	86.1 ± 0.1 ^b^	85.8 ± 0.3 ^b^	88.1 ± 0.1 ^a^	80.4 ± 0.6 ^d^	84.9 ± 0.1 ^c^
DDT (min)	7.76 ± 0.16 ^a^	5.79 ± 0.16 ^b^	4.55 ± 0.68 ^c^	5.91 ± 0.08 ^b^	5.43 ± 0.71 ^bc^	5.97 ± 0.16 ^b^	4.51 ± 0.69 ^c^	5.12 ± 0.00 ^bc^
ST (min)	10.15 ± 0.35 ^a^	5.10 ± 0.42 ^e^	4.15 ± 0.35 ^f^	6.75 ± 0.21 ^bc^	6.25 ± 0.21 ^bc^	6.85 ± 0.07 ^b^	5.25 ± 0.64 ^de^	6.00 ± 0.00 ^cd^
WD(C1-C2) (Nm)	0.59 ± 0.01 ^e^	0.71 ± 0.01 ^a^	0.68 ± 0.01 ^b^	0.62 ± 0.01 ^d^	0.68 ± 0.01 ^b^	0.63 ± 0.01 ^d^	0.63 ± 0.01 ^cd^	0.65 ± 0.01 ^c^
SV(C5-C4)(Nm)	0.98 ± 0.03 ^a^	0.51 ± 0.01 ^c^	0.58 ± 0.00 ^b^	0.50 ± 0.02 ^c^	0.44 ± 0.00 ^de^	0.41 ± 0.01 ^e^	0.48 ± 0.03 ^cd^	0.42 ± 0.00 ^e^

All values are mean ± SD, *n* = 3. Different letters in the same row indicate significant differences between parameters at the 0.05 level (*p* < 0.05).

**Table 3:** Effect of different pretreatments on the protein secondary structures of red kidney bean doughs (RKBDs).

Samples	Wheat Dough	Control	Peeling	Roasting	Atmospheric Steaming	High-Pressure Steaming	Sprouting	Microwaving
β-sheet (%)	31.12 ± 0.35 ^d^	36.35 ± 0.13 ^ab^	35.97 ± 0.25 ^ab^	36.52 ± 0.11 ^a^	33.39 ± 0.28 ^c^	32.94 ± 0.56 ^c^	35.89 ± 0.10 ^ab^	35.84 ± 0.02 ^b^
Irregular curl (%)	18.28 ± 0.22 ^a^	16.40 ± 0.08 ^bc^	16.45 ± 0.05 ^bc^	16.17 ± 0.04 ^c^	18.23 ± 0.17 ^a^	18.42 ± 0.21 ^a^	16.44 ± 0.06 ^bc^	16.50 ± 0.04 ^b^
α-helix (%)	18.27 ± 0.16 ^b^	17.42 ± 0.01 ^cd^	17.47 ± 0.10 ^c^	17.27 ± 0.06 ^d^	18.79 ± 0.02 ^a^	18.86 ± 0.08 ^a^	17.46 ± 0.06 ^cd^	17.44 ± 0.09 ^cd^
β-turn (%)	32.34 ± 0.42 ^a^	29.85 ± 0.20 ^bc^	30.11 ± 0.10 ^b^	30.05 ± 0.08 ^bc^	29.60 ± 0.09 ^c^	29.80 ± 0.26 ^bc^	30.22 ± 0.03 ^b^	30.23 ± 0.04 ^b^

All values are mean ± SD, *n* = 3. Different letters in the same row indicate significant differences between parameters at the 0.05 level (*p* < 0.05).

**Table 4:** Effect of different pretreatments on the baking attributes of red kidney bean doughs (RKBDs).

Samples	Wheat Bread	Control	Peeling	Roasting	Atmospheric Steaming	High-Pressure Steaming	Sprouting	Microwaving
Specific volume (cm^3^/g)	4.45 ± 0.07 ^a^	3.37 ± 0.04 ^c^	2.46 ± 0.03 ^e^	2.40 ± 0.07 ^ef^	3.53 ± 0.07 ^b^	3.29 ± 0.04 ^c^	2.31 ± 0.01 ^g^	2.90 ± 0.04 ^d^
Hardness (gf)	314.33 ± 17.04 ^h^	729.84 ± 23.87 ^f^	1651.90 ± 18.38 ^b^	1527.29 ± 23.8 ^c^	611.67 ± 2.58 ^g^	879.11 ± 15.29 ^e^	1732.13 ± 19.05 ^a^	929.58 ± 9.14 ^d^
Springiness	0.93 ± 0.01 ^a^	0.87 ± 0.01 ^d^	0.84 ± 0.01 ^e^	0.90 ± 0.00 ^c^	0.90 ± 0.01 ^bc^	0.91 ± 0.01 ^b^	0.79 ± 0.01 ^f^	0.88 ± 0.01 ^d^
Chewiness (gf)	189.67 ± 14.16 ^e^	352.42 ± 3.89 ^d^	821.75 ± 89.72 ^a^	849.02 ± 30.86 ^a^	386.75 ± 21.00 ^d^	518.59 ± 15.79 ^c^	689.69 ± 18.55 ^b^	530.85 ± 18.74 ^c^
Cohesiveness	0.65 ± 0.01 ^ab^	0.56 ± 0.03 ^cd^	0.59 ± 0.06 ^bc^	0.60 ± 0.02 ^bc^	0.70 ± 0.04 ^a^	0.65 ± 0.02 ^ab^	0.51 ± 0.01 ^d^	0.66 ± 0.03 ^a^
Resilience	0.31 ± 0.00 ^ab^	0.23 ± 0.01 ^c^	0.24 ± 0.03 ^c^	0.28 ± 0.02 ^b^	0.32 ± 0.02 ^a^	0.28 ± 0.01 ^b^	0.19 ± 0.01 ^d^	0.29 ± 0.00 ^ab^
Crust color								
L*	62.34 ± 0.62 ^a^	42.18 ± 0.58 ^e^	53.05 ± 1.29 ^b^	45.99 ± 0.34 ^d^	38.81 ± 1.29 ^f^	44.71 ± 0.64 ^d^	49.70 ± 0.29 ^c^	42.38 ± 1.22 ^e^
a*	9.67 ± 0.28 ^e^	16.69 ± 0.34 ^a^	13.07 ± 0.82 ^c^	14.15 ± 0.59 ^bc^	14.66 ± 0.44 ^b^	16.15 ± 0.69 ^a^	11.50 ± 1.09 ^d^	16.36 ± 0.42 ^a^
b*	30.40 ± 0.56 ^a^	23.07 ± 0.53 ^cd^	32.29 ± 0.90 ^a^	25.61 ± 1.36 ^b^	21.87 ± 1.30 ^d^	25.17 ± 1.60 ^bc^	23.65 ± 1.21 ^bcd^	23.21 ± 1.03 ^cd^
Whitening Index (WI)	50.64 ± 0.55 ^a^	35.54 ± 0.36 ^f^	41.52 ± 0.72 ^c^	38.57 ± 0.99 ^d^	33.38 ± 1.27 ^g^	37.13 ± 0.92 ^e^	43.23 ± 0.54 ^b^	35.75 ± 0.83 ^ef^
Browning Index (BI)	76.38 ± 1.91 ^b^	104.97 ± 0.49 ^a^	107.03 ± 1.62 ^a^	100.87 ± 7.76 ^a^	107.27 ± 8.43 ^a^	105.88 ± 7.94 ^a^	79.99 ± 5.41 ^b^	104.44 ± 0.68 ^a^
ΔE	-	22.57 ± 0.72 ^b^	10.12 ± 1.26 ^f^	17.65 ± 0.16 ^d^	25.55 ± 1.06 ^a^	19.54 ± 0.91 ^c^	14.49 ± 0.59 ^e^	22.25 ± 1.55 ^b^
Crumb color								
L*	66.62 ± 0.73 ^a^	55.25 ± 0.90 ^cd^	62.25 ± 0.65 ^b^	50.25 ± 0.75 ^e^	54.69 ± 1.09 ^cd^	47.65 ± 1.21 ^f^	56.20 ± 0.93 ^c^	53.95 ± 0.89 ^d^
a*	0.73 ± 0.02 ^e^	5.41 ± 0.23 ^bc^	1.52 ± 0.10 ^d^	5.83 ± 0.64 ^abc^	5.29 ± 0.15 ^c^	5.90 ± 0.26 ^ab^	6.07 ± 0.02 ^a^	5.91 ± 0.40 ^ab^
b*	9.52 ± 0.46 ^e^	10.68 ± 0.36 ^de^	14.57 ± 0.19 ^a^	13.69 ± 1.41 ^ab^	11.38 ± 0.75 ^cd^	12.60 ± 1.18 ^bc^	12.83 ± 0.64 ^bc^	11.66 ± 0.40 ^cd^
Whitening Index (WI)	65.27 ± 0.69 ^a^	53.68 ± 0.86 ^b^	65.03 ± 0.65 ^a^	48.06 ± 0.57 ^d^	52.97 ± 0.99 ^bc^	45.82 ± 0.92 ^ef^	53.95 ± 1.07 ^b^	52.12 ± 0.76 ^c^
Browning Index (BI)	15.88 ± 0.81 ^e^	28.28 ± 1.13 ^cd^	25.16 ± 0.65 ^d^	39.84 ± 4.19 ^a^	30.04 ± 1.82 ^bc^	39.32 ± 2.65 ^a^	33.46 ± 2.07 ^b^	31.96 ± 0.5 ^bc^
ΔE	-	12.35 ± 0.82 ^d^	5.4 ± 0.19 ^e^	17.69 ± 0.5 ^b^	12.93 ± 0.97 ^cd^	19.93 ± 0.96 ^a^	12.18 ± 0.97 ^d^	13.87 ± 0.74 ^c^

All values are mean ± SD, *n* = 3. Different letters in the same row indicate significant differences between parameters at the 0.05 level (*p* < 0.05).

**Table 5:** Effect of different pretreatments on the protein content and amino acid composition of red kidney bean bread (RKBB).

Samples	FAO (2013) [83]	Wheat Bread	Control	Peeling	Roasting	Atmospheric Steaming	High-Pressure Steaming	Sprouting	Microwaving
Protein content (dry, %)		16.85 ± 0.06 ^e^	29.43 ± 0.19 ^d^	30.96 ± 0.02 ^a^	29.94 ± 0.18 ^c^	29.57 ± 0.22 ^d^	29.88 ± 0.04 ^c^	30.19 ± 0.07 ^bc^	30.36 ± 0.01 ^b^
Thr	25	18.75 ± 0.07 ^f^	22.99 ± 0.15 ^b^	21.15 ± 0.01 ^e^	21.34 ± 0.13 ^e^	23.45 ± 0.17 ^a^	21.70 ± 0.03 ^d^	21.92 ± 0.05 ^cd^	21.95 ± 0.00 ^c^
Cys + Met	23	13.21 ± 0.05 ^bc^	13.09 ± 0.08 ^cd^	11.93 ± 0.01 ^g^	13.61 ± 0.08 ^a^	13.24 ± 0.10 ^b^	13.05 ± 0.02 ^d^	12.77 ± 0.03 ^e^	12.52 ± 0.00 ^f^
Val	40	38.96 ± 0.15 ^f^	45.83 ± 0.30 ^a^	42.72 ± 0.03 ^e^	43.80 ± 0.26 ^d^	45.27 ± 0.34 ^b^	43.76 ± 0.06 ^d^	44.48 ± 0.10 ^c^	42.66 ± 0.01 ^e^
Ile	30	29.93 ± 0.11 ^f^	32.42 ± 0.21 ^a^	30.29 ± 0.02 ^e^	30.93 ± 0.19 ^d^	32.02 ± 0.24 ^b^	31.24 ± 0.04 ^d^	31.62 ± 0.07 ^c^	31.11 ± 0.01 ^d^
Leu	61	58.77 ± 0.22 ^d^	61.54 ± 0.40 ^a^	57.91 ± 0.04 ^e^	58.75 ± 0.35 ^d^	61.45 ± 0.46 ^a^	59.70 ± 0.08 ^c^	60.58 ± 0.13 ^b^	58.86 ± 0.01 ^d^
Phe + Tyr	41	69.69 ± 0.27 ^e^	74.38 ± 0.48 ^ab^	70.12 ± 0.05 ^e^	71.05 ± 0.43 ^d^	74.87 ± 0.56 ^a^	72.51 ± 0.10 ^c^	73.70 ± 0.16 ^b^	71.44 ± 0.01 ^d^
Lys	48	19.44 ± 0.07 ^e^	32.71 ± 0.21 ^a^	30.76 ± 0.02 ^d^	30.73 ± 0.19 ^d^	32.59 ± 0.24 ^a^	32.13 ± 0.04 ^b^	32.90 ± 0.07 ^a^	31.66 ± 0.01 ^c^
Trp	6.6	5.24 ± 0.02 ^e^	5.32 ± 0.03 ^d^	5.99 ± 0.00 ^a^	5.58 ± 0.03 ^b^	5.48 ± 0.04 ^c^	4.51 ± 0.01 ^f^	3.95 ± 0.01 ^g^	3.59 ± 0.00 ^h^
EAA/TAA (%)		30.01 ± 0.11 ^b^	34.11 ± 0.52 ^a^	34.04 ± 0.05 ^a^	34.15 ± 0.48 ^a^	33.58 ± 0.59 ^a^	33.67 ± 0.11 ^a^	33.98 ± 0.17 ^a^	33.82 ± 0.02 ^a^
EAAI (%)		43.68 ± 0.17 ^e^	50.02 ± 0.32 ^a^	47.71 ± 0.03 ^c^	48.57 ± 0.29 ^b^	50.26 ± 0.37 ^a^	47.69 ± 0.07 ^c^	47.32 ± 0.10 ^c^	45.75 ± 0.01 ^d^

All values are mean ± SD, *n* = 3. Different letters in the same row indicate significant differences between parameters at the 0.05 level (*p* < 0.05).

**Table 6:** Effect of different pretreatments on the digestive characteristics of red kidney bean bread (RKBB).

Samples	Wheat Bread	Control	Peeling	Roasting	Atmospheric Steaming	High-Pressure Steaming	Sprouting	Microwaving
Total starch (%)	59.97 ± 0.4 ^a^	40.91 ± 0.28 ^c^	40.58 ± 1.84 ^c^	41.11 ± 0.79 ^c^	43.25 ± 0.54 ^b^	41.22 ± 0.18 ^c^	41.20 ± 0.39 ^c^	41.04 ± 0.18 ^c^
C_∞_ (%)	88.28 ± 0.17 ^a^	76.95 ± 0.19 ^d^	78.09 ± 0.43 ^c^	78.86 ± 0.24 ^b^	74.49 ± 0.16 ^e^	77.38 ± 0.16 ^cd^	76.97 ± 0.61 ^d^	75.02 ± 0.05 ^e^
K	0.064 ± 0.000 ^a^	0.053 ± 0.001 ^c^	0.056 ± 0.000 ^b^	0.054 ± 0.001 ^c^	0.063 ± 0.000 ^a^	0.062 ± 0.000 ^a^	0.050 ± 0.000 ^d^	0.057 ± 0.001 ^b^
R^2^	0.9878	0.9940	0.9917	0.9892	0.9915	0.9926	0.9952	0.9925
eGI	94.40 ± 0.16 ^a^	82.05 ± 0.39 ^c^	83.39 ± 0.40 ^b^	84.00 ± 0.15 ^b^	80.64 ± 0.15 ^d^	83.56 ± 0.20 ^b^	81.12 ± 0.60 ^d^	80.75 ± 0.07 ^d^
RDS (%)	64.91 ± 0.08 ^a^	51.97 ± 0.81 ^d^	53.64 ± 0.26 ^c^	53.64 ± 0.10 ^c^	53.86 ± 0.09 ^c^	56.16 ± 0.28 ^b^	48.99 ± 0.45 ^e^	52.55 ± 0.40 ^d^
SDS (%)	23.34 ± 0.10 ^c^	24.85 ± 0.59 ^b^	24.37 ± 0.17 ^b^	25.10 ± 0.33 ^b^	20.60 ± 0.06 ^e^	21.18 ± 0.11 ^e^	27.79 ± 0.15 ^a^	22.41 ± 0.45 ^d^
RS (%)	11.76 ± 0.17 ^e^	23.18 ± 0.21 ^b^	22.00 ± 0.43 ^cd^	21.27 ± 0.23 ^d^	25.55 ± 0.16 ^a^	22.67 ± 0.16 ^bc^	23.23 ± 0.61 ^b^	25.05 ± 0.04 ^a^

All values are mean ± SD, *n* = 3. Different letters in the same row indicate significant differences between parameters at the 0.05 level (*p* < 0.05).

## Data Availability

The original contributions presented in this study are included in the article. Further inquiries can be directed to the corresponding author.
